# Fracture of Rib, Followed by Extensive Tissue Emphysema

**Published:** 1891-01

**Authors:** C. D. Roy

**Affiliations:** House Surgeon Charity Hospital, New York


### FRACTURE OF RIB, FOLLOWED BY EXTENSIVE TISSUE EMPHYSEMA.

BY C. D. ROY, A. B. M. D., HOUSE SURGEON CHARITY HOSPITAL, N. Y.

This article is presented to the readers of THE RECORD, not on account of its exceeding rarity, but on account of the infrequency of such extensive tissue emphysema and such distinct lines of demarkation. Before remarking upon the case and its pathological conditions, I will give in detail the history of the patient who was under my charge.

John W., age 35, laborer, was admitted into the hospital on October 18th, 1890. His family history gave negative results, while his previous history resulted as follows: Had the small pox thirty years ago; has worked the most of his life as a longshoreman, during which time he has never been sick; has never been much of a drinker up to three weeks ago, and during the last three days previous to his entrance into the hospital he was in a continuous state of intoxication. The latter condition is the cause of the majority of acute surgical cases which enter these charitable institutions. Of his present history, he informed me that on the night of the 17th of October, while under the influence of liquor, he had fallen down one of the hatch-ways along the sidewalk, sustaining some severe wrenches of the body. Although not rendered unconscious by the fall, he was unable to move, and had to be taken in an ambulance to Chambers Street Hospital. At the latter place a diagnosis of fractured rib was made, surgical assistance rendered, and the next day he was transferred to this hospital, and to my ward. Upon being called to the ward, I made a superficial examination, as it was now late at night, he suffering no pain and the dressing in no way needing attention. The next morning, however, I made a thorough examination, and found the following conditions present: Inspection and palpation showed a fracture of the tenth rib at its angle. There was distinct crepetation, the patient complaining of much pain at the seat of lesion, and a feeling as if something was giving away at that point. There was a contused wound in the superior palpebral region on the left side, and quite a severe ecchymosis of the lids on the right. There were no contusions on chest wall, nor any sign of external injury at the seat of fracture. The patient appeared nervous, due to his late alcoholic excesses, but in other respects presenting a well nourished body. The left side of the body, from the pelvis up to the inferior maxilla, presented the appearance of puffiness. Respiratory movements were somewhat decreased, due to the injured rib and the pain occasioned by the movement of the fragments.

Percussion gave the marked pneumothorax sound on the affected side; that is, a peculiar box-tone resonance. On striking the side with the palm of the hand, the peculiar sound of cushion puffiness could be elicited. By pressing gently with the finger tips upon the cutaneous surface, marked crackling sounds, or rather, sensations of compressing air, were readily recognized. To my mind the nearest approach to the sensation is that elicited by placing the tongue against the anterior portion of the hard palate, and then compressing the air as it is forced out through the two surfaces. The crepitation was limited behind by the spinal column up to the sixth cervical vertebra, and anteriorly it only extended across the median line from the ensiform cartilage to symphysis pubes. It was present throughout the neck, but not marked posteriorly; limited below by pouparts ligament and the crest of the ilium; above by the inferior maxilla and a line drawn from its angle backward. There was no abnormal displacement of internal viscera. Complaining somewhat of dysphagia and laryngeal oppression, an examination was made of these parts, resulting, however, in negative results, save the morbid changes usually found in a sub-acute laryngitis. On auscultation no marked abnormal condition was noticed, and strange to say, the respiratory murmur was fairly clear throughout the attack. Temperature and pulse normal, but respirations somewhat increased, due to the pain occasioned by a full inspiration, thus irritating the already injured pleura. His appetite was good and nightly rest in no way disturbed, except when lying upon the affected side, causing pressure of the lung upon the seat of injury.

October 20th. Patient's dysphagia much lessened, and scarcely any pain around the throat. Crepitus still markedly prominent over the areas first mentioned and pain at seat of injury.

October 21st. Crepitus decreasing, as also the pain and tenderness. Appetite good and the patient walks around the ward without difficulty.

October 22d. Crepitus disappeared from the neck and around the shoulder, diminishing the area.

October 23d. Crepitation is now only present within a radius of four inches from sèat of injury. Percussion still gives the marked pneumothorax sound.

October 29th. Emphysema scarcely perceptible in the tissues, but pain during the respiratory movements is still present.

November 3d. All crepitation has entirely disappeared. Still complains of the dry pleuretic stitch.

November 7th. Patient can now do heavy lifting around the ward. Complains of scarcely any pain.

November 15th. Patient discharged. Adhesive plaster ordered to be kept around the chest for two weeks.

The case in question having proved so unique in character, I determined to seek further information on the subject by consulting those authorities who have made mention of it in their works. No writer have I found who has devoted any space to its consideration, the majority simply mentioning it as an occasional sequella in fractured rib. Fracture of the rib usually occurs either (1) directly as from a fall or a blow at the point of injury; or (2) indirectly, as when the pressure is transmitted from a blow received upon some adjacent structure. For the sake of convenience I have classified two varieties of tissue emphysema—that occurring with and that without an external opening. Whenever a rib is fractured and there results an emphysema of the surrounding tissues, it necessarily implies the wounding of the lungs, pleura and subcostal fascia, except in those rare cases where there is rupture of an air vesicle within the lung and subsequent emphysema of the neck, a condition of which I shall make mention further on.

In comparison with the above, I wish to mention another case of the same condition, but more severe and rapid in its result. The case occurred upon the surgical division of the hospital, and it is through the kindness of the house surgeon that I am enabled to report it.

Charles F., Swede, æt. 27, was admitted into the hospital December 12th, with the diagnosis of fractured rib. He died so soon after his entrance into the building that no definite facts could be gathered by the surgeon in charge, and hence the history is necessarily meagre. On the morning of the same day, while working upon one of the piers of East River, he lost his hold and was precipitated some distance, falling upon his right side and back. An ambulance was summoned, the patient picked up and carried to Chambers Street Hospital. There his side was encased in adhesive strips and the patient, evidently feeling much better, he was transferred to Charity Hospital. Arriving upon the four o'clock boat, he walked into the office and furnished the usual information. As he was turning away from the desk, he began to be cyanotic, and at the same time fell upon the floor. The surgeon was quickly called, but found all endeavors to restoration futile, and in ten minutes the patient was dead.

On objective examination there was found an ecchymosis over the seat of fracture, but no external opening. Extensive tissue (cellular) emphysema of the whole body was found from the feet to the crown of the head. No part was exempt, for the sub-costal opening was evidently so large that an indefinite volume of air was continually passing out into the tissues. Finally the air had accumulated to such an extent that the pressure in the pleural cavity was equal to that in the lungs, and hence came the collapse of that organ. No post mortem was made, but the pathological conditions could be readily conjectured from the objective and subjective symptoms. The two cases are unique, and placed together furnish an excellent type upon which a prognosis may be based.

Pneumothorax, or accumulation of air into the pleural cavity, is the usual accompaniment of emphysema of the tissues, though Erichsen says they may both occur independently of each other. Of the two forms of this pathological condition, that occurring with an external opening is the more common, especially when the latter is of small dimensions. When pneumothorax alone develops as a result of an injured rib, the visceral layer of the pleura and the lung itself are alone perforated, unless the perforation of the costal layer is so small as to prevent the escape of any air at that point. But, when the tissue at all four points are injured, the lungs, two layers of pleura and sub-costal fascia, the resulting emphysema occurs as follows: With each inspiration all the air vesicles of the lungs are filled, and with each expiration the air is driven out from the lungs through the nose and mouth by way of the bronchi, and in case of injury, into the pleural cavity, by far the largest volume escaping through the former passage. The pressure on the pleural cavity being negative, in health it does not take long for the pressure to be so increased as to cause the air in that cavity to seek some point of escape. This exit being furnished through the injured sub-costal fascia, with each expiration a certain amount of air escapes into the areolar tissue of the chest wall around the seat of injury. The greater the expansibility of the lung tissue, the greater will be the pressure upon the air vesicles during expiration, and hence the greater pressure to aid in the escape of the already accumulating air in the pleural cavity. As a usual thing, the air in the tissues around the seat of injury do not extend out in a radius of more than several inches. In the present case the air escaped as far superficially as the deep fascia covering the muscles of the trunk. This deep fascia is continuous with that in the neck up to the body of the inferior maxilla and inferior curved line of the occipital bone, behind and below the crest of the ilium, forms the posterior boundary, while the intimate relationship of this fascia with the structures attached to pouparts ligament, forms the anterior boundary below. Thus the tissue emphysema was found to be confined in these anatomical limits, and wherever the fasciæ seemed to be closely blended together or to that of the adjacent structures, there the pathological condition ceased. Hinton, quoted by Erichsen, speaks of an emphysematous state in the neck due to the rupture of an air vesicle without any external injury, the air escaping through the mediastina, along the sheath of the blood vessels into the neck. The injury to the chest wall in the present case was fully capable of accounting for the emphysema of such wide area without conjecturing in regard to the escape of the air through the channels of the great vessels.

The prognosis in these cases is nearly always favorable, the air in the majority of cases being absorbed without any injurious effects.

The symptoms are well exemplified by the history of the case hereby presented, and I have found that no disease is so well understood as those represented by illustrative cases.

Nothing can be said of the treatment, save that it is carried out symptomatically. Firm circular compression around the trunk with rubber adhesive strips have given the best results, putting, as it were, the chest wall in splints and at the same time affording resistance to the escape of air from the pleural cavity. For emphysema of the neck, I found that sheet lint dipped in equal parts of sweet oil and oil of turpentine, applied around the neck with a firm bandage compression, was very efficacious in its result. The sparseness of the literature upon this subject has led me to make these few remarks, in hopes that it may not be entirely uninteresting to those who have and those who have not seen such cases.

